# Efficacy of Bacille Calmette–Guérin Against COVID-19 Hospitalisation: A Meta-Analysis and Systematic Review of Randomised Control Trials

**DOI:** 10.3390/vaccines13030267

**Published:** 2025-03-04

**Authors:** Joseph Inauen, Sam LaBroome, Antonietta Maldari, Natalie E. Stevens, James B. Geake, David J. Lynn, Simone Barry

**Affiliations:** 1 Royal Adelaide Hospital, Port Road, Adelaide, SA 5000, Australia; 2 Faculty of Health and Medical Sciences, University of Adelaide, North Terrace, Adelaide, SA 5000, Australia; 3 Precision Medicine Theme, South Australian Health and Medical Research Institute, North Terrace, Adelaide, SA 5000, Australia; natalie.stevens@sahmri.com (N.E.S.); david.lynn@sahmri.com (D.J.L.); 4 Flinders Health and Medical Research Institute, College of Medicine and Public Health, Flinders University, Bedford Park, SA 5042, Australia

**Keywords:** BCG, COVID-19, SARS-CoV-2, systematic review, meta-analysis

## Abstract

Background: The BCG vaccine has long been hypothesised to have non-specific protective effects, and early epidemiological studies on COVID-19 suggested a possible protective effect against SARS-CoV-2 infection and COVID-19 severity. This systematic review and meta-analysis assesses the effect of the BCG vaccine on preventing severe COVID-19 disease, based on the rate of hospitalisation for COVID-19 related disease. Methods: We performed a literature search of randomised control trials comparing BCG vaccine to placebo in adult participants using EMBASE, MEDLINE, and Web of Science. A random effects model was used to generate summary estimates. Risk of bias was assessed regarding randomisation, allocation sequence concealment, blinding, incomplete outcome data, selective outcome reporting, and other biases. Results: We included 11 studies involving 18,412 participants, reporting COVID-19 incidence. The hospitalisation rate was sought from the authors of papers that did not report on this statistic. There was no significant reduction in COVID-19-related hospitalisation across all studies (relative risk 0.85, 0.51–1.40, *p* = 0.335), COVID-19 incidence across all studies (relative risk 1.07, 0.94–1.21, *p* = 0.264), deaths reported in six studies (relative risk 0.67, 0.36–1.26, *p* = 0.733), and COVID-19-related critical care admissions reported in four studies (relative risk 0.43, 0.13–1.46, *p* = 0.746). Conclusions: The findings from this meta-analysis, involving a large number of participants, suggest no protective effect of BCG vaccination against severe COVID-19 outcomes or overall SARS-CoV-2 incidence. Further research may be needed to explore the potential non-specific effects of BCG vaccination in other specific populations and against other infections.

## 1. Introduction 

The COVID-19 pandemic was unprecedented in its rapid global spread, and there was a clear early need for effective strategies to prevent or attenuate infection. Prior to the introduction of COVID-19 specific vaccines, there was considerable interest in the use of the Bacillus Calmette–Guérin (BCG) vaccine for off-target protection against COVID-19. Given discrepancies regarding the BCG vaccine’s effect on COVID-19 and the unclear clinical benefits of trained immunity, this meta-analysis aims to better understand the role of the BCG vaccine’s off-target effects in the prevention of COVID-19. 

The BCG vaccine has been used for over a century for the prevention of tuberculosis disease. In recent decades, there has been interest in the off-target immunomodulatory effects of the BCG vaccine, thought to be mediated via trained immunity [1], a process that is hypothesised to be responsible for reduced all-cause mortality in children vaccinated with BCG [1,2,3,4,5]. Early cohort, epidemiological, and ecological data on the COVID-19 pandemic suggested possible protective effects of the BCG vaccine against COVID-19, as disease burden and mortality were lower in regions where BCG vaccination is prevalent [6,7,8,9,10,11,12,13,14,15,16,17,18]. However, multiple confounding factors may have influenced these observations including the stage of the epidemic, age structure of countries with BCG vaccination policies, and potential under-diagnosis or under-reporting of COVID-19 in low- and middle-income countries where BCG vaccination is widespread [8,19]. Experimental in vitro data suggest that BCG vaccination reduces cytokine production and increases viral clearance in whole blood stimulated with irradiated SARS-CoV-2, which may result in reduced disease severity [20,21]. 

In the search for an effective control measure for the global pandemic, many trials looking at the effectiveness of BCG vaccination against COVID-19 were conducted. A previous systematic review and meta-analysis of randomised controlled trials reported no significant effect of BCG vaccination on COVID-19 incidence or severity [22]. However, subsequent studies have significantly increased the number of participants included in the analysis, offering a broader dataset for robust statistical interpretation. This systematic review and meta-analysis of randomised controlled trials aims to determine the effect of BCG vaccination, compared to placebo, on the rate of COVID-19-related hospitalisation, as an indirect measure of disease severity. 

## 2. Methods

We conducted a systematic review complying with the methods described in the PRISMA statement to report systematic reviews and meta-analyses [23]. The protocol for the review was registered in the PROSPERO International Prospective Register of Systematic Reviews (registration number CRD42024581424).

### 2.1. Data Sources and Searches

A literature search was performed using EMBASE, MEDLINE, and Web of Science. The search strategy was designed to include all eligible trials, including randomised control trials looking at the effect of BCG vaccination on COVID-19 disease from 1 January 2019 up until the search date 15 March 2024. 

The detailed search strategy for MEDLINE follows: (“COVID-19”[Mesh] OR “SARS-CoV-2”[mesh] OR (“coronavirus”[mesh:noexp] AND 2019/01/01:2024/03/15[dp]) OR (“coronavirus infections”[mesh] AND 2019/01/01:2024/03/15[dp]) OR 2019 novel coronavirus[tiab] OR 2019 nCoV[tiab] OR 2019-nCoV[tiab] OR 2019nCoV[tiab] OR coronavirus disease 2[tiab] OR coronavirus disease 2019[tiab] OR Coronavirus Disease 19[tiab] OR coronavirus disease-19[tiab] OR coronavirus infection* 2019[tiab] OR COVID[tiab] OR COVID-19[tiab] OR COVID19[tiab] OR COVID2019[tiab] OR nCoV 2019[tiab] OR nCoV2019[tiab] OR novel coronavirus 2019[tiab] OR novel coronavirus disease 2019[tiab] OR novel coronavirus infection* 2019[tiab] OR paucisymptomatic coronavirus disease 2019[tiab] OR SARS coronavirus 2[tiab] OR SARS CoV 2[tiab] OR SARS-CoV-2[tiab] OR SARS-CoV2[tiab] OR SARSCoV2[tiab] OR severe acute respiratory syndrome 2[tiab] OR severe acute respiratory syndrome coronavirus 2[tiab] OR severe acute respiratory syndrome coronavirus 2019[tiab] OR severe acute respiratory syndrome CoV-2[tiab] OR Wuhan coronavirus[tiab])AND (“BCG Vaccine”[Mesh] OR (“Mycobacterium bovis”[Mesh] AND “Vaccines”[Mesh]) OR BCGbacile Calmette Guerin[tiab] OR bacille Calmette Guerin[tiab] OR bacilli Calmette Guerin[tiab] OR bacillus Calmette Guerin[tiab] OR bacillus de Calmette Guerin[tiab] OR bacillus of Calmette Guerin[tiab] OR Calmette Guerin bacille[tiab] OR Calmette Guerin bacilli[tiab] OR calmette guerin bacillus[tiab] OR M. bovis Bacillus Calmette Guerin[tiab] OR Calmette’s Vaccine[tiab] OR Calmette Vaccine[tiab] OR Calmettes Vaccine[tiab]).

The detailed search strategy for the other sources can be found in the Supplementary Material. The results were transferred to EndNote and, finally, Covidence for duplicate removal and additional evaluation. 

### 2.2. Study Selection

Two independent reviewers screened all titles and abstracts to select studies for further analysis, excluding irrelevant articles. Two authors reviewed the full text of the remaining articles independently to ensure the study met all inclusion criteria. A third reviewer was consulted for any ambiguity.

Inclusion criteria required placebo-controlled randomised control trials with BCG vaccination as the intervention, and COVID-19 specific reported outcomes confirmed by genotypic or serological testing. Non-human studies, non-randomised control trials, those without a placebo-control group, and studies that did not test for COVID-19 infection were excluded. Papers published in languages other than English were also excluded.

The primary outcome was the severity of COVID-19 based on hospitalisation due to COVID-19 disease. If studies did not report a COVID-19-related hospitalisation rate, these data were sought directly from the investigators of those studies before inclusion in this analysis. Secondary outcomes included COVID-19 incidence, COVID-19-associated critical care admissions, and mortality. 

Where available, data on prior BCG vaccination history (whether administered in childhood or adulthood) were extracted from the included studies. Six of the eleven included studies reported prior BCG vaccination rates, which ranged from 17% (The Netherlands) to 88% (Brazil). However, prior BCG vaccination was not an inclusion or exclusion criterion, and studies did not consistently stratify outcomes based on participants’ vaccination history. Given the potential immunomodulatory effects of prior BCG exposure, we conducted additional statistical analyses to assess whether this factor influenced our findings.

### 2.3. Data Extraction and Quality Assessment

Two independent investigators extracted data from each study. The extracted data included first author’s name, year of publication, country, participant characteristics, intervention and control data, outcome data, and funding sources. All data were entered into Covidence for consistent management and to identify discrepancies between reviewers. 

The RoB2 tool [24] was used to assess the risk of bias for individual studies across five domains: randomisation process, deviations from intended interventions, missing outcome data, measurement of the outcome, and selection of the reported result. Two independent assessors evaluated each study, with a third assessor available to resolve disagreements. All studies, independent of their risk of bias, were included in the outcome analysis. 

The Grading of Recommendations Assessment, Development, and Evaluation (GRADE) [25] recommendations were used to assess the quality of evidence of each outcome, based on risk of bias, consistency of results, directness of evidence, and precision. 

### 2.4. Statistical Analysis 

All statistical analyses were carried out using Stata version 17 (StataCorp. 2023. Stata Statistical Software: Release 17. College Station, TX, USA: StataCorp LLC). Dichotomous outcomes were reported as frequencies with an effect size measured by risk ratios (RRs) with confidence intervals (CIs), displayed in forest plots. A random effects model was used for statistical analysis. Statistical heterogeneity between studies was calculated using I^2^ statistics for all outcomes. Funnel plots were used to assess publication bias. 

To assess the potential impact of prior BCG vaccination history, we conducted additional analyses. Heterogeneity was first assessed across all studies using Cochran’s Q test, I^2^ statistic, and τ^2^ estimates to evaluate overall between-study variability. Following this, studies were stratified into high (>50%) and low (<50%) prior BCG exposure groups, and heterogeneity was re-evaluated within each subgroup to determine whether differences in prior BCG vaccination contributed to variability in the effect estimates.

Furthermore, we conducted a meta-regression analysis to examine whether the proportion of participants with prior BCG vaccination in each study influenced the pooled effect size. Additionally, a subgroup analysis was performed comparing studies with high (>50%) vs. low (<50%) prior BCG vaccination rates. Finally, a leave-one-out sensitivity analysis was conducted to evaluate whether the exclusion of individual studies, including those using modified BCG formulations (e.g., VPM1002 [26]), significantly altered the overall findings.

## 3. Results 

### 3.1. Search Results 

**Figure 1:**
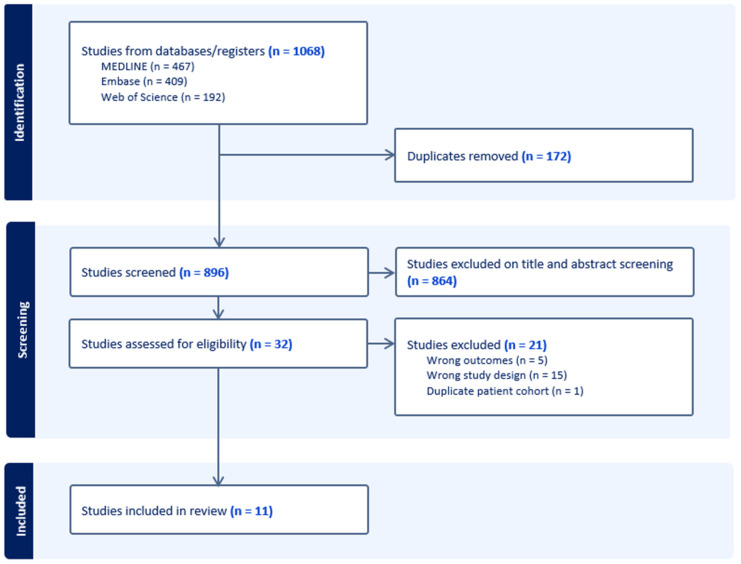
Flowchart of the literature search and included studies.

The search identified 1068 studies of potential relevance, of which 172 duplicates were removed (Figure 1). The remaining 896 were screened by title and abstract resulting in the exclusion of 864 due to irrelevance. A total of 32 studies underwent full text review, and 21 were excluded for the following reasons: wrong outcome (n = 5), inappropriate study design (n = 15), or duplicate patient cohort (n = 1). Ultimately, 11 studies, comprising over 18,000 participants, were included in the analysis. 

A PRISMA flow diagram depicting the selection process for studies included in the meta-analysis. The figure details the number of records identified, screened, excluded, and included in the final analysis

### 3.2. Study Characteristics 

Participant demographics were well balanced between the intervention and control groups across all studies. However, patient characteristics varied between studies due to differences in study populations (Table 1). Four studies enrolled only older participants [26,27,28,29], while two studies included participants with comorbidities [29,30]. Six studies focussed on health care workers, comprising a younger cohort with a high proportion of females [31,32,33,34,35,36]. Six studies reported prior BCG vaccination rates, ranging from 17% in the Netherlands to 88% in Brazil. 

Ten of the eleven studies were conducted across multiple sites, including one multinational study [32] spanning five countries: Australia, Netherlands, Spain, the United Kingdom, and Brazil. All studies the number of COVID-19-related hospitalisations or provided these data upon request from a study investigator. The follow-up period was at least six months in all studies. 

### 3.3. Primary Outcome 

**Figure 2:**
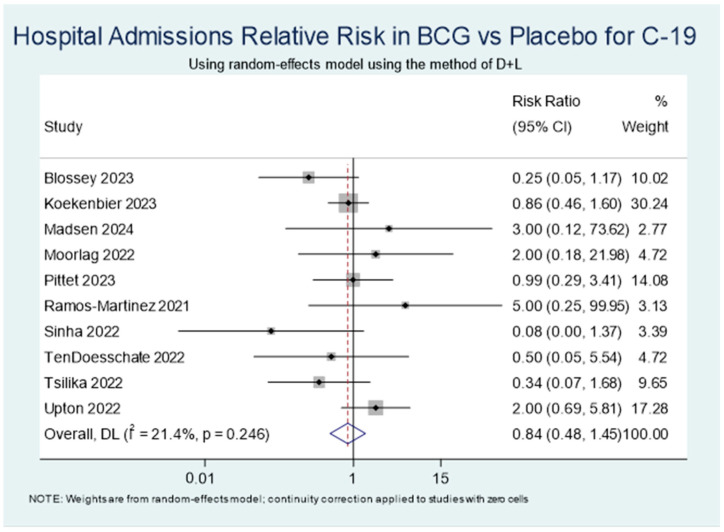
Primary outcome—forest plot of COVID-19-related hospital admissions. Blossey 2023 [26], Koekenbier 2023 [27], Madsen 2024 [31], Moorlag 2022 [28], Pittet 2023 [32], Ramos-Martinez 2021 [33], Santos 2023 [34], Sinha 2022 [30], TenDoesschate 2022 [35], Tsilika 2022 [29], Upton 2022 [36].

A total of 18,412 participants from the 11 included studies were analysed in the meta-analysis. There was no significant difference in the rate of COVID-19-related hospitalisation between the BCG and placebo control groups (RR 0.84, 95% CI 0.48–1.45) using a random-effects model for statistical analysis to account for study variability (Figure 2). Low heterogeneity was observed among included studies (I^2^ = 21.4%, *p* = 0.246). 

A leave-one-out sensitivity analysis (Table 2) showed the combined effect estimate remains 0.85 (95% CI: 0.51–1.40) across study omissions, suggesting that no single study overly influenced the meta-analysis. Blossey et al. [26], which evaluated a genetically modified BCG strain VPM1002, had the largest impact, where exclusion slightly shifted the pooled estimate (RR = 0.96, 95% CI: 0.61–1.51), although it did not meaningfully alter conclusions. The exclusion of Upton et al. [36] resulted in a lower estimate of 0.72 (95% CI: 0.45–1.17), suggesting it has a stronger signal toward risk reduction.

### 3.4. Secondary Outcomes 

**Figure 3:**
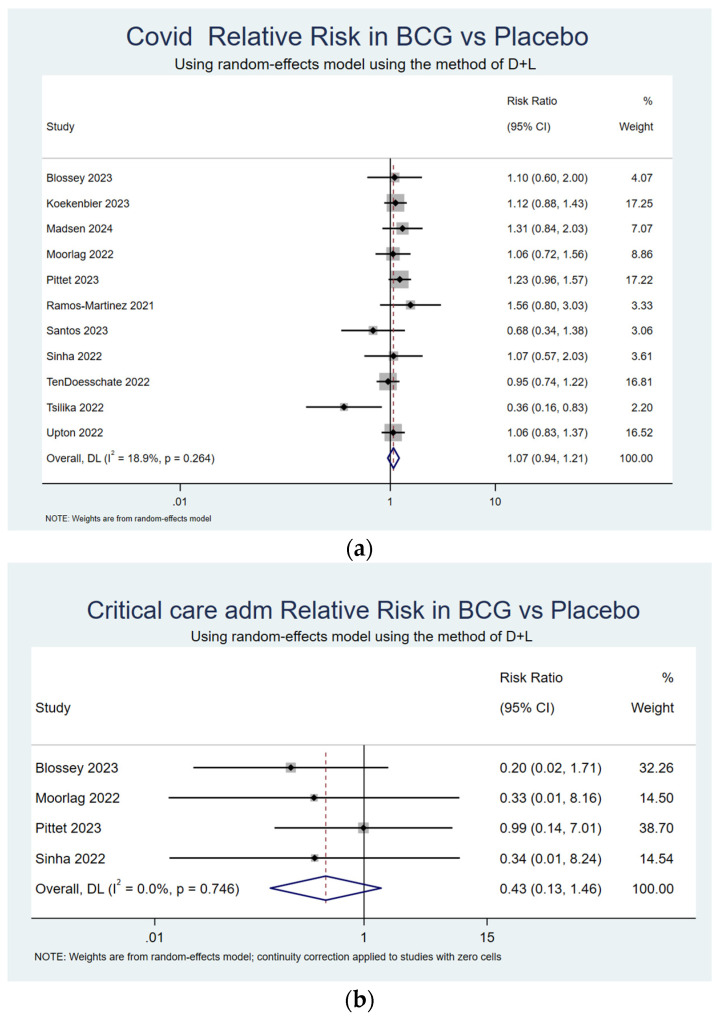

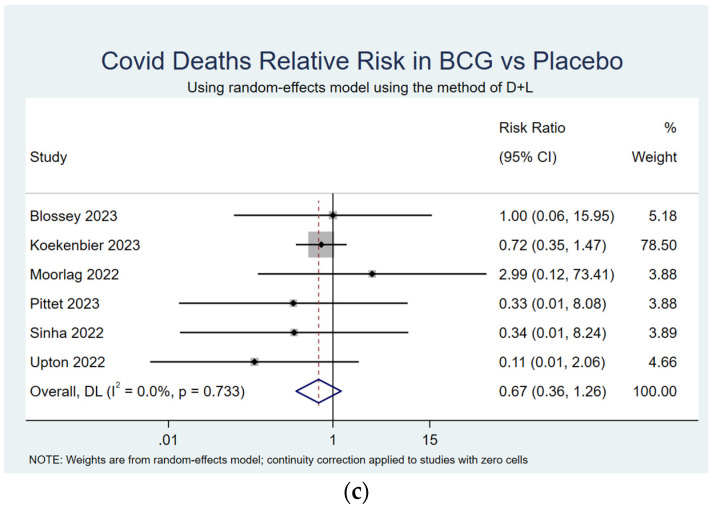
Secondary outcomes—forest plots: (**a**) COVID-19 incidence; (**b**) COVID-19-related critical care admissions; (**c**) COVID-19 deaths. Forest plots depicting the relative risk (RR) and 95% confidence intervals (CI) for secondary outcomes. The random-effects model was used for analysis. Heterogeneity between studies was assessed using the I^2^ statistic. Blossey 2023 [26], Koekenbier 2023 [27]Madsen 2024 [31], Moorlag 2022 [28], Pittet 2023 [32], Ramos-Martinez 2021 [33], Santos 2023 [34], Sinha 2022 [30], TenDoesschate 2022 [35], Tsilika 2022 [29], Upton 2022 [36].

The incidence of COVID-19 was reported in all the included studies, and a random-effects model was again used to assess for intergroup differences. There was no statistically significant difference in COVID-19 rates between the BCG vaccination and placebo control groups (RR 1.07, 95% CI 0.94–1.21) with low heterogeneity between studies (I^2^ = 18.9%, *p* = 0.264) (Figure 3a). 

The rate of COVID-19-associated critical care admissions, reported in 4 of the 11 studies was not statistically different between the BCG vaccination and placebo groups (RR 0.43, 95% CI 0.13–1.46, I^2^ 0.0%, *p* = 0.746) (Figure 3b). Similarly, there was no significant difference in COVID-19-related mortality between the intervention and control groups (RR 0.67, 95% CI 0.36–1.26, I^2^ 0.0, *p* 0.733) (Figure 3c). 

### 3.5. Quality Assessment 

Nine of the eleven studies were assessed as having a low risk of bias using Cochrane’s RoB2 assessment tool. One study was rated as having some concerns due to issues with the randomisation process and selection of the reported result (Table 3). Another study was deemed high risk of bias due to outcome measurement, where a statistically significant difference was observed in the self-reporting rate of results between the intervention and control groups. 

**Figure 4:**
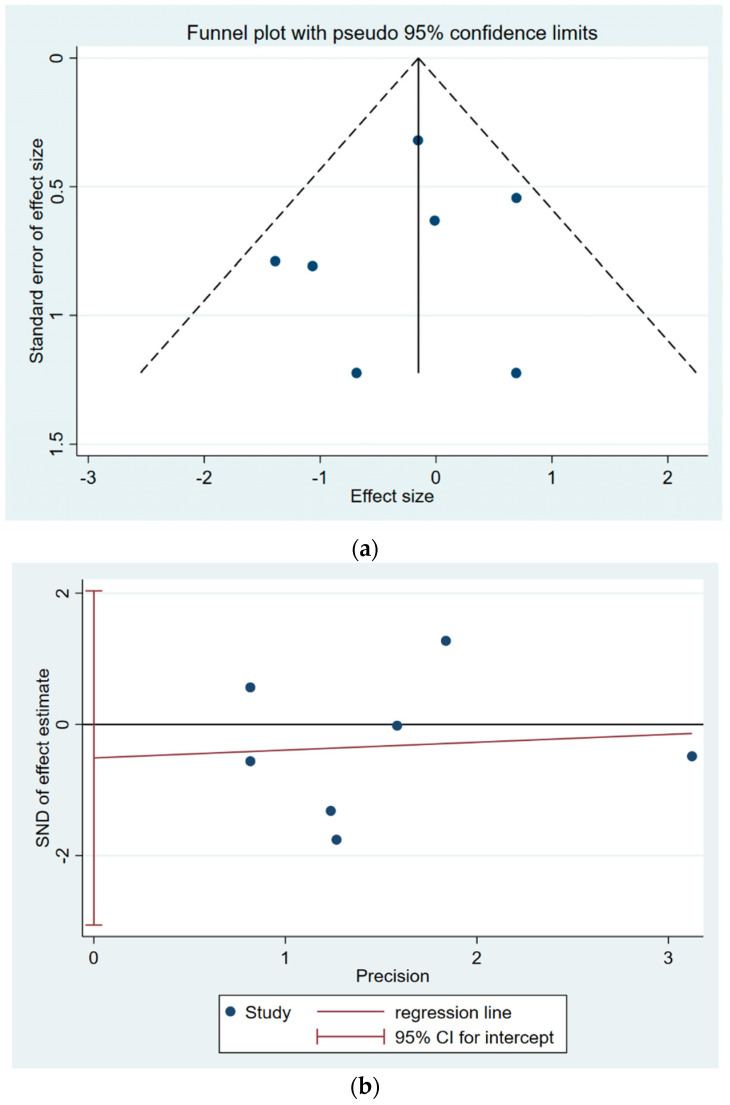
(**a**): Funnel plot for COVID-19–related hospitalisations. A funnel plot assessing potential publication bias in studies reporting COVID-19–related hospitalisation. Symmetry suggests low risk of publication bias. (**b**): Eggers test for COVID-19–related hospitalisation. Egger’s regression test evaluating the presence of publication bias in studies reporting COVID-19-related hospitalisation. A *p*-value of 0.628 indicates low risk of bias.

For the primary outcome of COVID-19-related hospitalisation, no significant publication bias was detected based on the visual inspection of the funnel plot (Figure 4a) and Eggers test (*p* = 0.628), indicating low risk of publication bias (Figure 4b). 

### 3.6. GRADE Assessment

The GRADE approach was used to evaluate the quality of evidence for each primary and secondary outcome across the four domains: risk of bias, consistency of results, directness of evidence, and precision. The quality of evidence was assessed as ‘high’ for all domains across all outcomes. 

### 3.7. Assessing Potential Impact of Prior BCG Vaccination History

To assess the potential impact of prior BCG vaccination, we stratified studies into two groups based on reported prior BCG vaccination rates: high prior BCG vaccination (>50%) and low prior BCG vaccination (<50%). Heterogeneity analysis was then performed separately for each group. The overall I^2^ statistic (12.5%, *p* = 0.335) and Cochran’s Q test (Q = 6.85, df = 6, *p* = 0.335) indicated minimal between-study variability, suggesting that differences in prior BCG vaccination were unlikely to be a major confounder. The heterogeneity variance (τ^2^ = 0.0601) was also low, reinforcing the consistency of findings.

Meta-regression analysis testing the effect of prior BCG vaccination rates did not identify a significant association (*p* = 0.47), suggesting that variations in prior vaccination history among study participants did not meaningfully impact the risk ratio estimates.

Subgroup analysis comparing studies with high (>50%) vs. low (<50%) prior BCG vaccination rates found no significant differences between groups. 

## 4. Discussion 

This meta-analysis of 11 RCTs, encompassing 18,412 participants, found no significant reduction in COVID-19-related hospitalisation following BCG vaccination compared to placebo. Secondary outcomes, including critical care admission, mortality, and COVID-19 incidence, similarly showed no significant differences between groups. These findings challenge early epidemiological and ecological studies that suggested a protective effect of BCG vaccination against SARS-CoV-2, emphasising the need for rigorous clinical trials to substantiate hypotheses generated from observational data.

### 4.1. Comparison to Prior Research and the Role of Trained Immunity

Several previous studies have investigated the potential effects of BCG against COVID-19 with varying conclusions. Early population-level studies [8,17,37] suggested an inverse correlation between BCG vaccination policies and COVID-19 burden, leading to the hypothesis that trained immunity induced by BCG could confer broad antiviral protection. However, observational studies are prone to confounding factors, including differences in health care infrastructure, demographic distributions, and pandemic control measures. 

Trained immunity is a process of reprogramming innate immune cells [38,39], to develop a heightened response to subsequent infections following an initial stimulus. Unlike adaptive immunity, which relies on memory B and T cells to generate specific immune responses, trained immunity is mediated by innate immune cells such as monocytes, macrophages, and natural killer (NK) cells. This process is driven by epigenetic and metabolic reprogramming of innate immune cells and their progenitors, leading to an enhanced response. 

A 2022 meta-analysis incorporating only observational studies reported a protective effect of BCG on SARS-CoV-2 outcomes [40]. However, a subsequent 2023 meta-analysis by Wen et al. included nine RCTs with just under 8000 participants and found no significant effect of BCG on COVID-19 incidence and severity. Our study builds on this by incorporating additional large-scale trials, more than doubling the number of participants, and reinforcing the conclusion that BCG vaccination does not significantly alter the risk of severe COVID-19 disease.

### 4.2. Study Strengths

The analysis benefited from several strengths. The inclusion of only placebo controlled RCTs ensures a high standard of evidence, reducing the impact of selection and reporting bias that may have affected earlier observational studies. The multinational and multicentre nature of the included trials enhances the generalizability of findings across different populations. Furthermore, the low statistical heterogeneity (I^2^ < 25% across all outcomes) increases confidence in the robustness of these findings.

Unlike prior meta-analyses that included heterogeneous study designs, our review focused exclusively on RCTs that explicitly reported COVID-19-related hospitalisation or provided these data upon request, making hospitalisation a reliable proxy for disease severity. 

### 4.3. Limitations and Potential Confounders

Despite these strengths, several limitations should be acknowledged. Firstly, variability in the study populations may mask a true effect size in subpopulations. For example, individual studies in our analysis enrolled only young health care workers, while others enrolled only older individuals or those with high comorbidity index. It remains unclear whether specific subgroups, such as immunocompromised individuals, might derive benefit from BCG vaccination. Future studies should conduct subgroup analyses to explore these potential differential effects.

Variation in BCG strains used across studies presents a potential confounder. Multiple BCG strains with differing immunogenic profiles were utilised, and it is possible that some strains elicit stronger trained immunity responses than others. While some studies suggest that BCG strain differences influence immune priming [41], there is no clear evidence that any particular strain provides non-specific protection against COVID-19.

Furthermore, Blossey et al. [26] evaluated VPM1002, a genetically modified BCG preparation rather than the conventional BCG vaccine. While VPM1002 retains key immunostimulatory properties of BCG, it may have distinct effects on trained immunity, potentially influencing study outcomes. To assess whether this study introduced bias, we conducted a sensitivity analysis excluding Blossey 2023, which resulted in a slightly higher pooled estimate (RR = 0.96, 95% CI: 0.61–1.51) but did not meaningfully alter the overall conclusions. This suggests that, while strain variation may contribute to minor heterogeneity, it does not significantly impact the findings.

Another potential limitation is the variation in prior BCG vaccination history among study populations. Six of the eleven included studies reported prior BCG vaccination rates, but these were not consistently recorded across all trials. Given that prior BCG exposure could influence trained immunity, we conducted additional analyses to assess its impact.

Heterogeneity analysis (I^2^ = 12.5%) and Cochran’s Q test (*p* = 0.335) suggested minimal between-study variability, indicating that prior BCG exposure was unlikely to be a major confounder. Meta-regression analysis (*p* = 0.47) further confirmed that the proportion of previously vaccinated participants did not significantly alter the pooled effect estimate. Additionally, a subgroup analysis comparing high (>50%) vs. low (<50%) prior BCG exposure groups found no meaningful difference in effect sizes.

Taken together, these findings suggest that while prior BCG exposure could theoretically influence trained immunity, it did not have a statistically significant impact on this meta-analysis’ conclusions. Future studies may benefit from explicitly stratifying participants by prior BCG vaccination history to better assess potential immune priming effects. 

SARS-CoV-2-specific vaccinations became widely available during the follow-up period of the included studies. The potential interaction between BCG-induced trained immunity and the adaptive immune response to COVID-19 vaccines remains unclear. Some studies have hypothesised that BCG may enhance responses to subsequent SARS-CoV-2 vaccines [18,42], as seen with influenza [43,44], although no difference was seen between BCG and placebo groups despite COVID-19 vaccination.

The overall event rate for the primary outcome was low, limiting the power of the analysis. Although hospitalisation, critical care admissions, and mortality were objectively recorded, self-reported data were used in several studies for COVID-19 incidence and mild disease outcomes. The reliance on self-reported outcomes introduces the potential for response bias. While our quality assessment indicated that 9 of the 11 studies had a low risk of bias, future studies should aim for standardised and objective outcome reporting to minimise bias further.

While the methodological rigour of this review minimises bias, there are inherent limitations in the review process itself. The exclusion of non-English studies may have led to the omission of relevant data. Additionally, although study selection and data extraction were conducted independently by multiple reviewers, potential discrepancies in interpretation remain a limitation of any systematic review. Future reviews should consider broader language inclusion and further validation methods.

### 4.4. Implications for Practice, Policy, and Future Research

The findings of this meta-analysis have important implications for the understanding of BCG-induced trained immunity and its clinical relevance. While BCG has been shown to induce broad immune modulation and has demonstrated protective effects against respiratory infections in some studies [45,46,47,48,49], its role in preventing severe viral diseases remains uncertain. Our findings do not support the use of BCG vaccination as a strategy to reduce severe COVID-19 outcomes in the general adult population.

From a policy perspective, these findings indicate that resources should not be allocated to BCG as a COVID-19 preventive measure. Instead, future pandemic preparedness efforts should focus on evidence-based interventions, such as targeted vaccination strategies, antiviral development, and immunomodulatory therapies with clearer benefits [50,51]. The lack of significant protective effects also suggests that further research into BCG’s broader non-specific effects should be carefully designed to focus on conditions where trained immunity may play a more pronounced role.

Future research should explore specific populations that may still benefit from BCG-induced trained immunity, noting especially the strong evidence of BCG’s protective effect against all-cause mortality in infants. Subgroup analyses of older adults, individuals with prior BCG exposure, or those with underlying immunosuppression may yield different findings. Additionally, long-term studies investigating whether BCG-induced immune training provides protection against other respiratory pathogens, such as influenza or RSV, could further clarify the broader clinical significance of trained immunity. Further meta-analyses incorporating emerging RCTs will be necessary to refine our understanding of these effects over time.

## 5. Conclusions

This study does not support the use of BCG to prevent severe COVID-19 in the general adult population. These findings reinforce the importance of large-scale high-quality RCTs in accessing vaccine efficacy beyond observational associations. The results contribute to the ongoing investigation into the clinical significance of trained immunity thought to be provided by the BCG vaccine against off-target pathogens. Further research is required to investigate the potential clinical significance of trained immunity from BCG vaccination beyond COVID-19. 

## Figures and Tables

**Table 1:** **Study characteristics**. Summary of key characteristics of the 11 included studies, including participant demographics, intervention details, and study design. VPM1002 = recombinant BCG vaccine candidate deficient in urease and expressing listeriolysin. N/A = not available.

Study	Country	Intervention BCG Strain	Participants	Female (%)	Age—Median (IQR) or Mean	Cardiovascular Disease	Hypertension	Chronic Respiratory Disease	Smoker	Diabetes	Previous BCG Vaccination
**Blossey 2023 [26]**	Germany	VPM1002	2025	47.1%	66 (60–91)	N/A		N/A	N/A	N/A	N/A
**Koekenbier 2023 [27]**	Netherlands	Danish strain 1331	6112	37.2%	69 (65–74)	413 (7%)	3477 (57%)	1642 (27%)	N/A	1040 (17%)	908 (15%)
**Madsen 2024 [31]**	Denmark	Danish strain 1331	1230	82.3%	47 (36–57)	70 (6%)	N/A	61 (5%)	171 (14%)	13 (1%)	651 (53%)
**Moorlag 2022 [28]**	Netherlands	Danish Strain 1331	2014	47.5%	67 (64–72)	370 (18%)	616 (31%)	186 (9%)	102 (5%)	137 (7%)	550 (27%)
**Pittet 2023 [32]**	Multinational	Denmark	3386	63.3%	42	447 (13%)		203 (6%)	360 (11%)	119 (4%)	2506 (74%)
**Ramos-Martinez 2021 [33]**	Mexico	Pasteur Meriux Connaught	60	88.3%	38 (28–54)	N/A		N/A	N/A	N/A	N/A
**Santos 2023 [34]**	Brazil	Moreau strain or Moscow strain	278	75.5%	N/A	N/A	31 (11%)	6 (2%)	24 (9%)	6 (2%)	246 (88%)
**Sinha 2022 [30]**	India	N/A	495	47.9%	43	163 (33%)	N/A	76 (15%)	N/A	250 (51%)	N/A
**TenDoesschate 2022 [35]**	Netherlands	Danish strain 1331	1511	74.3%	42	34 (2%)	99 (7%)	133 (9%)	123 (8%)	9 (1%)	256 (17%)
**Tsilika 2022 [29]**	Greece	Moscow strain 361-I	301	32.2%	68	42 (14%)	87 (29%)	73 (24%)	N/A	62 (21%)	N/A
**Upton 2022 [36]**	South Africa	Danish strain 1331	1000	70.4%	39 (30–50)	24 (2%)	174 (17%)	72 (7%)	274 (27%)	63 (6%)	N/A

**Table 2:** **Leave-one-out sensitivity analysis.** The combined effect estimate remains 0.85 (95% CI: 0.51–1.40) across study omissions.

Study Omitted	Estimate	95% Confidence Interval	
**Blossey 2023 [26]**	0.956	0.606	1.508
**Koekenbier 2023 [27]**	0.803	0.382	1.687
**Madsen 2024 [31]**	0.846	0.512	1.397
**Moorlag 2022 [28]**	0.803	0.463	1.391
**Pittet 2023 [32]**	0.801	0.427	1.503
**Ramos-Martinez 2021 [33]**	0.846	0.512	1.397
**Santos 2023 [34]**	0.846	0.512	1.397
**Sinha 2022 [30]**	0.846	0.512	1.397
**TenDoesschate 2022 [35]**	0.856	0.485	1.509
**Tsilika 2022 [29]**	0.927	0.561	1.533
**Upton 2022 [36]**	0.724	0.448	1.169
**Combined**	**0.846**	**0.512**	**1.397**

**Table 3:** **Quality assessment using Risk of Bias (RoB2) Tool**. Assessment of the risk of bias across the five domains using Cochrane’s RoB2 tool. Studies were classified overall as low risk, some concerns, or high risk of bias.

RoB2	Randomisation Process	Deviations from Intended Interventions	Missing Outcome Data	Measurement of the Outcome	Selection of the Reported Results	Overall
**Blossey 2023 [26]**	Low	Low	Low	Low	Low	Low
**Koekenbier 2023 [27]**	Low	Low	Low	Low	Low	Low
**Madsen 2024 [31]**	Low	Low	Low	High	Low	High
**Moorlag 2022 [28]**	Low	Low	Low	Low	Low	Low
**Pittet 2023 [32]**	Low	Low	Low	Low	Low	Low
**Ramos-Martinez 2021 [33]**	Low	Low	Low	Low	Low	Low
**Santos 2023 [34]**	Low	Low	Low	Low	Low	Low
**Sinha 2022 [30]**	Low	Low	Low	Low	Low	Low
**TenDoesschate 2022 [35]**	Low	Low	Low	Low	Low	Low
**Tsilika 2022 [29]**	Some Concerns	Low	Low	Low	Some Concerns	Some Concerns
**Upton 2022 [36]**	Low	Low	Low	Low	Low	Low

## Data Availability

All data extracted from included studies and used in analyses are available upon request from the corresponding author. The analytic code and data extraction templates are also available upon request.

## Supplementary Materials

The following supporting information can be downloaded at: https://www.mdpi.com/article/10.3390/vaccines13030267/s1, Supplementary Table S1: Search Terms; Supplementary Table S2: Complete Search Strategy.
