# Supplementary material for: Efficacy of Bacille Calmette–Guérin Against COVID-19 Hospitalisation: A Meta-Analysis and Systematic Review of Randomised Control Trials
Source: Vaccines (Basel). 2025 Mar 4;13(3):267. doi: 10.3390/vaccines13030267 (PMC11945481; doi:10.3390/vaccines13030267)
Supplement: Supplementary file 1 [file vaccines-13-00267-s001.zip › vaccines-3481475-supplementary.pdf]

Supplementary Material: Efficacy of bacille Calmette-Guérin against COVID-19 hospitalisation: a meta-analysis and systematic review of randomised control trials.

Supplementary Table 1: Search Terms

	MeSH (Pubmed) Terms	Embase (Ovid) Terms	Synonyms
**Concept 1**<br><br>**BCG**	"BCG Vaccine"[Mesh]<br><br>("Mycobacterium bovis"[Mesh] AND "Vaccines"[Mesh])	mycobacterium bovis bcg/	BCG<br>bacile Calmette Guerin<br>bacille Calmette Guerin<br>bacilli Calmette Guerin<br>bacillus Calmette Guerin<br>bacillus de Calmette Guerin<br>bacillus of Calmette Guerin<br>bcg copenhagen 1331<br>Calmette Guerin bacille<br>Calmette Guerin bacilli<br>calmette guerin bacillus<br>M. bovis Bacillus Calmette Guerin<br>Calmette's Vaccine<br>Calmette Vaccine<br>Calmettes Vaccine
**Concept 2**<br><br>**COVID**	"COVID-19"[Mesh]<br>"SARS-CoV-2"[mesh]<br><br>("coronavirus"[mesh:noexp] AND 2019/01/01:2024/03/15[dp])<br><br>("coronavirus infections"[mesh] AND 2019/01/01:2024/03/15[dp])	Exp coronavirus disease 2019/<br><br>exp Severe acute respiratory syndrome coronavirus 2/	2019 novel coronavirus<br>2019 nCoV<br>2019-nCoV<br>2019nCoV<br>coronavirus disease 2<br>coronavirus disease 2019<br>Coronavirus Disease 19<br>coronavirus disease-19<br>coronavirus infection* 2019<br>COVID<br>COVID-19<br>COVID19<br>COVID2019<br>nCoV 2019<br>nCoV2019<br>novel coronavirus 2019<br>novel coronavirus disease 2019<br>novel coronavirus infection* 2019<br>paucisymptomatic coronavirus disease 2019<br>SARS coronavirus 2<br>SARS CoV 2<br>SARS-CoV-2<br>SARS-CoV2<br>SARSCoV2<br>severe acute respiratory syndrome 2<br>severe acute respiratory syndrome coronavirus 2<br>severe acute respiratory syndrome coronavirus 2019<br>severe acute respiratory syndrome CoV-2<br>Wuhan coronavirus

Supplementary Table 2: Complete Search Strategy

Pubmed	("COVID-19"[Mesh] OR "SARS-CoV-2"[mesh] OR ("coronavirus"[mesh:noexp] AND 2019/01/01:2024/03/15[dp]) OR ("coronavirus infections"[mesh] AND 2019/01/01:2024/03/15[dp]) OR 2019 novel coronavirus[tiab] OR 2019 nCoV[tiab] OR 2019-nCoV[tiab] OR 2019nCoV[tiab] OR coronavirus disease 2[tiab] OR coronavirus disease 2019[tiab] OR Coronavirus Disease 19[tiab] OR coronavirus disease-19[tiab] OR coronavirus infection* 2019[tiab] OR COVID[tiab] OR COVID-19[tiab] OR COVID19[tiab] OR COVID2019[tiab] OR nCoV 2019[tiab] OR nCoV2019[tiab] OR novel coronavirus 2019[tiab] OR novel coronavirus disease 2019[tiab] OR novel coronavirus infection* 2019[tiab] OR paucisymptomatic coronavirus disease 2019[tiab] OR SARS coronavirus 2[tiab] OR SARS CoV 2[tiab] OR SARS-CoV-2[tiab] OR SARS-CoV2[tiab] OR SARSCoV2[tiab] OR severe acute respiratory syndrome 2[tiab] OR severe acute respiratory syndrome coronavirus 2[tiab] OR severe acute respiratory syndrome coronavirus 2019[tiab] OR severe acute respiratory syndrome CoV-2[tiab] OR Wuhan coronavirus[tiab]) AND ("BCG Vaccine"[Mesh] OR ("Mycobacterium bovis"[Mesh] AND "Vaccines"[Mesh]) OR BCG bacile Calmette Guerin[tiab] OR bacille Calmette Guerin[tiab] OR bacilli Calmette Guerin[tiab] OR bacillus Calmette Guerin[tiab] OR bacillus de Calmette Guerin[tiab] OR bacillus of Calmette Guerin[tiab] OR Calmette Guerin bacille[tiab] OR Calmette Guerin bacilli[tiab] OR calmette guerin bacillus[tiab] OR M. bovis Bacillus Calmette Guerin[tiab] OR Calmette's Vaccine[tiab] OR Calmette Vaccine[tiab] OR Calmettes Vaccine[tiab])
Embase (Ovid)	https://salus.idm.oclc.org/login?url=http://ovidsp.ovid.com/ovidweb.cgi?T=JS&NEWS=N&PAGE=main&SHAREDSEARCHID=5OZeEmsimMknl88eHl5wn35eh8GQ5NgS5bJhSS0dlpXg90zVoDBdxCSJ9fsMDeHVU<br><br>(Exp coronavirus disease 2019/<br>OR<br>exp Severe acute respiratory syndrome coronavirus 2/<br>OR<br>2019 novel coronavirus.ti,ab,kf<br>2019 nCoV.ti,ab,kf<br>2019-nCoV.ti,ab,kf<br>2019nCoV.ti,ab,kf<br>coronavirus disease 2.ti,ab,kf<br>coronavirus disease 2019.ti,ab,kf<br>Coronavirus Disease 19.ti,ab,kf<br>coronavirus disease-19.ti,ab,kf<br>coronavirus infection* 2019.ti,ab,kf<br>COVID.ti,ab,kf<br>COVID-19.ti,ab,kf<br>COVID19.ti,ab,kf<br>COVID2019.ti,ab,kf<br>nCoV 2019.ti,ab,kf<br>nCoV2019.ti,ab,kf<br>novel coronavirus 2019.ti,ab,kf<br>novel coronavirus disease 2019.ti,ab,kf<br>novel coronavirus infection* 2019.ti,ab,kf<br>paucisymptomatic coronavirus disease 2019.ti,ab,kf<br>SARS coronavirus 2.ti,ab,kf<br>SARS CoV 2.ti,ab,kf<br>SARS-CoV-2.ti,ab,kf<br>SARS-CoV2.ti,ab,kf<br>SARSCoV2.ti,ab,kf<br>severe acute respiratory syndrome 2.ti,ab,kf<br>severe acute respiratory syndrome coronavirus 2.ti,ab,kf<br>severe acute respiratory syndrome coronavirus 2019.ti,ab,kf<br>severe acute respiratory syndrome CoV-2.ti,ab,kf<br>Wuhan coronavirus.ti,ab,kf<br>)<br>AND<br>(<br>exp mycobacterium bovis bcg/<br>OR<br>BCG.ti,ab,kf<br>bacile Calmette Guerin.ti,ab,kf<br>bacille Calmette Guerin.ti,ab,kf<br>bacilli Calmette Guerin.ti,ab,kf<br>bacillus Calmette Guerin.ti,ab,kf

	bacillus de Calmette Guerin.ti,ab,kf<br>bacillus of Calmette Guerin.ti,ab,kf<br>Calmette Guerin bacille.ti,ab,kf<br>Calmette Guerin bacilli.ti,ab,kf<br>calmette guerin bacillus.ti,ab,kf<br>bovis Bacillus Calmette Guerin.ti,ab,kf<br>Calmette's Vaccine.ti,ab,kf<br>Calmette Vaccine.ti,ab,kf<br>Calmettes Vaccine.ti,ab,kf)
Web of Science	(TI=("BCG" OR "bacile Calmette Guerin" OR "bacille Calmette Guerin" OR "bacilli Calmette Guerin" OR "bacillus Calmette Guerin" OR "bacillus de Calmette Guerin" OR "bacillus of Calmette Guerin" OR "bcg copenhagen 1331" OR "BCG live" OR "Calmette Guerin bacilli" OR "Calmette Guerin bacilli" OR "calmette guerin bacillus" OR "M. bovis Bacillus Calmette Guerin" OR "M. bovis BCG" OR "mycobacterium bcg" OR "Mycobacterium bovis var. BCG" OR "Mycobacterium tuberculosis bovis BCG" OR "Mycobacterium tuberculosis var. bovis BCG" OR "Calmette's Vaccine" OR "Calmette Vaccine" OR "Calmettes Vaccine") OR AB=("BCG" OR "bacile Calmette Guerin" OR "bacille Calmette Guerin" OR "bacilli Calmette Guerin" OR "bacillus Calmette Guerin" OR "bacillus de Calmette Guerin" OR "bacillus of Calmette Guerin" OR "bcg copenhagen 1331" OR "BCG live" OR "Calmette Guerin bacilli" OR "Calmette Guerin bacilli" OR "calmette guerin bacillus" OR "M. bovis Bacillus Calmette Guerin" OR "M. bovis BCG" OR "mycobacterium bcg" OR "Mycobacterium bovis var. BCG" OR "Mycobacterium tuberculosis bovis BCG" OR "Mycobacterium tuberculosis var. bovis BCG" OR "Calmette's Vaccine" OR "Calmette Vaccine" OR "Calmettes Vaccine") OR AK=("BCG" OR "bacile Calmette Guerin" OR "bacille Calmette Guerin" OR "bacilli Calmette Guerin" OR "bacillus Calmette Guerin" OR "bacillus de Calmette Guerin" OR "bacillus of Calmette Guerin" OR "bcg copenhagen 1331" OR "BCG live" OR "Calmette Guerin bacilli" OR "Calmette Guerin bacilli" OR "calmette guerin bacillus" OR "M. bovis Bacillus Calmette Guerin" OR "M. bovis BCG" OR "mycobacterium bcg" OR "Mycobacterium bovis var. BCG" OR "Mycobacterium tuberculosis bovis BCG" OR "Mycobacterium tuberculosis var. bovis BCG" OR "Calmette's Vaccine" OR "Calmette Vaccine" OR "Calmettes Vaccine")) AND (TI=("2019 novel coronavirus disease" OR "2019 novel coronavirus epidemic" OR "2019 novel coronavirus infection*" OR "2019 nCoV Disease" OR "2019 nCoV Infection*" OR "2019-nCoV disease" OR "2019-nCoV infection*" Or "coronavirus disease 2" OR "coronavirus disease 2019" OR "Coronavirus Disease 19" OR "coronavirus disease-19" OR "coronavirus infection* 2019" OR "COVID" OR "COVID 19" OR "COVID 2019" OR "COVID-19" OR "COVID19" OR "COVID-19 Virus Infection*" OR "COVID 19 Virus Infection*" OR "COVID-19 Virus Disease" OR "COVID 19 Virus Disease" OR "nCoV 2019 disease" OR "nCoV 2019 infection*" OR "novel coronavirus 2019 disease" OR "novel coronavirus 2019 infection*" OR "novel coronavirus disease 2019" OR "novel coronavirus infection* 2019" OR "paucisymptomatic coronavirus disease 2019" OR "SARS coronavirus 2 infection*" OR "SARS CoV 2 Infection*" OR "SARS-CoV-2 disease" OR "SARS-CoV-2 infection*" OR "SARS-CoV2 disease" OR "SARS-CoV2 infection*" OR "SARSCoV2 disease" OR "SARSCoV2 infection*" OR "severe acute respiratory syndrome 2" OR "severe acute respiratory syndrome coronavirus 2 infection*" OR "severe acute respiratory syndrome coronavirus 2019 infection*" OR "severe acute respiratory syndrome CoV-2 infection*" OR "Wuhan coronavirus disease" OR "Wuhan coronavirus infection*") OR AB=("2019 novel coronavirus disease" OR "2019 novel coronavirus epidemic" OR "2019 novel coronavirus infection*" OR "2019 nCoV Disease" OR "2019 nCoV Infection*" OR "2019-nCoV disease" OR "2019-nCoV infection*" Or "coronavirus disease 2" OR "coronavirus disease 2019" OR "Coronavirus Disease 19" OR "coronavirus disease-19" OR "coronavirus infection* 2019" OR "COVID" OR "COVID 19" OR "COVID 2019" OR "COVID-19" OR "COVID19" OR "COVID-19 Virus Infection*" OR "COVID 19 Virus Infection*" OR "COVID-19 Virus Disease" OR "COVID 19 Virus Disease" OR "nCoV 2019 disease" OR "nCoV 2019 infection*" OR "novel coronavirus 2019 disease" OR "novel coronavirus 2019 infection*" OR "novel coronavirus disease 2019" OR "novel coronavirus infection* 2019" OR "paucisymptomatic coronavirus disease 2019" OR "SARS coronavirus 2 infection*" OR "SARS CoV 2 Infection*" OR "SARS-CoV-2 disease" OR "SARS-CoV-2 infection*" OR "SARS-CoV2 disease" OR "SARS-CoV2 infection*" OR "SARSCoV2 disease" OR "SARSCoV2 infection*" OR "severe acute respiratory syndrome 2" OR "severe acute respiratory syndrome coronavirus 2 infection*" OR "severe acute respiratory syndrome coronavirus 2019 infection*" OR "severe acute respiratory syndrome CoV-2 infection*" OR "Wuhan coronavirus disease" OR "Wuhan coronavirus infection*") OR AK=("2019 novel coronavirus disease" OR "2019 novel coronavirus epidemic" OR "2019 novel coronavirus infection*" OR "2019 nCoV Disease" OR "2019 nCoV Infection*" OR "2019-nCoV disease" OR "2019-nCoV infection*" Or "coronavirus disease 2" OR "coronavirus disease 2019" OR "Coronavirus Disease 19" OR "coronavirus disease-19" OR "coronavirus infection* 2019" OR "COVID" OR "COVID 19" OR "COVID 2019" OR "COVID-19" OR "COVID19" OR "COVID-19 Virus Infection*" OR "COVID 19 Virus Infection*" OR "COVID-19 Virus Disease" OR "COVID 19 Virus Disease" OR "nCoV 2019 disease" OR "nCoV 2019 infection*" OR "novel coronavirus 2019 disease" OR "novel coronavirus 2019 infection*" OR "novel coronavirus disease 2019" OR "novel coronavirus infection* 2019" OR

	"paucisymptomatic coronavirus disease 2019" OR "SARS coronavirus 2 infection*" OR "SARS CoV 2 Infection*" OR "SARS-CoV-2 disease" OR "SARS-CoV-2 infection*" OR "SARS-CoV2 disease" OR "SARS-CoV2 infection*" OR "SARSCoV2 disease" OR "SARSCoV2 infection*" OR "severe acute respiratory syndrome 2" OR "severe acute respiratory syndrome coronavirus 2 infection*" OR "severe acute respiratory syndrome coronavirus 2019 infection*" OR "severe acute respiratory syndrome CoV-2 infection*" OR "Wuhan coronavirus disease" OR "Wuhan coronavirus infection*"))
